# μ-2,3,5,6-Tetra-2-pyridylpyrazine-κ^3^ *N* ^1^,*N* ^2^,*N* ^6^:κ^3^ *N* ^3^,*N* ^4^,*N* ^5^-bis­[(methanol-κ*O*)(nitrato-κ^2^ *O*,*O*′)(nitrato-κ*O*)cadmium(II)]

**DOI:** 10.1107/S1600536808022320

**Published:** 2008-07-19

**Authors:** Mirabdullah Seyed Sadjadi, Amin Ebadi, Karim Zare, Vahid Amani, Hamid Reza Khavasi

**Affiliations:** a Department of Chemistry, Science and Research Campus, Islamic Azad University, Poonak, Tehran, Iran; b Department of Chemistry, Shahid Beheshti University, Evin, Tehran 1983963113, Iran; c Department of Chemistry, Islamic Azad University, Shahr-e-Rey Branch, Tehran, Iran

## Abstract

The title complex, [Cd_2_(NO_3_)_4_(C_24_H_16_N_6_)(CH_4_O)_2_], displays a centrosymmetric dinuclear structure, in which the 2,3,5,6-tetra-2-pyridinylpyrazine (tppz) ligand links two Cd ions separated by 7.323 (4) Å. Each Cd^II^ center is seven-coordinated by three N-atom donors of tppz in one plane, by two O atoms nearly normal to this plane, and by two O atoms 0.393 (3) and 0.488 (3) Å from that plane. The two Cd^II^ ions are above and below the plane of the pyrazine ring of the tppz ligand, oriented with respect to the pyridine rings at dihedral angles of 38.01 (3) and 31.90 (3)°. The dihedral angle between the two pyridine rings is 41.11 (3)°. In the crystal structure, inter­molecular O—H⋯O hydrogen bonds link the mol­ecules.

## Related literature

For related literature, see: Bock *et al.* (1992 ▶); Carranza *et al.* (2004 ▶); Goodwin & Lyons (1959 ▶); Graf *et al.* (1993 ▶, 1997 ▶); Greaves & Stoeckli-Evans (1992 ▶); Hadadzadeh *et al.* (2006 ▶); Laine *et al.* (1995 ▶); Sakai & Kurashima (2003 ▶); Yamada *et al.* (2000 ▶); Zhang *et al.* (2005 ▶).
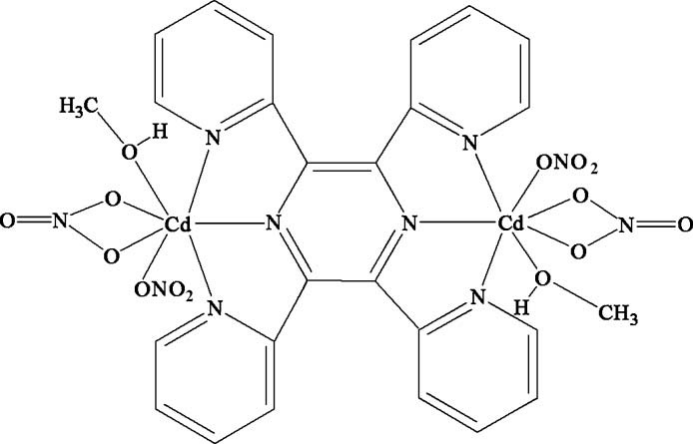


## Experimental

### 

#### Crystal data

[Cd_2_(NO_3_)_4_(C_24_H_16_N_6_)(CH_4_O)_2_]
*M* *_r_* = 925.37
Monoclinic, 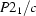

*a* = 9.0777 (12) Å
*b* = 10.8949 (9) Å
*c* = 16.690 (2) Å
β = 93.847 (10)°
*V* = 1646.9 (3) Å^3^
*Z* = 2
Mo *K*α radiation
μ = 1.38 mm^−1^
*T* = 298 (2) K
0.50 × 0.40 × 0.25 mm

#### Data collection

Stoe IPDSII diffractometer
Absorption correction: numerical (*X-SHAPE*; Stoe & Cie, 2005 ▶) *T*_min_ = 0.510, *T*_max_ = 0.710
4664 measured reflections
4642 independent reflections
4223 reflections with *I* > 2σ(*I*)
*R*_int_ = 0.075

#### Refinement

*R*[*F* ^2^ > 2σ(*F* ^2^)] = 0.062
*wR*(*F* ^2^) = 0.171
*S* = 1.07
4642 reflections
239 parameters
H atoms treated by a mixture of independent and constrained refinement
Δρ_max_ = 1.00 e Å^−3^
Δρ_min_ = −0.95 e Å^−3^

### 

Data collection: *X-AREA* (Stoe & Cie, 2005 ▶); cell refinement: *X-AREA*; data reduction: *X-RED* (Stoe & Cie, 2005 ▶); program(s) used to solve structure: *SHELXS97* (Sheldrick, 2008 ▶); program(s) used to refine structure: *SHELXL97* (Sheldrick, 2008 ▶); molecular graphics: *ORTEP-3 for Windows* (Farrugia, 1997 ▶); software used to prepare material for publication: *WinGX* (Farrugia, 1999 ▶).

## Supplementary Material

Crystal structure: contains datablocks I, global. DOI: 10.1107/S1600536808022320/hk2497sup1.cif

Structure factors: contains datablocks I. DOI: 10.1107/S1600536808022320/hk2497Isup2.hkl

Additional supplementary materials:  crystallographic information; 3D view; checkCIF report

## Figures and Tables

**Table:** 

O1—Cd1	2.330 (4)
O2—Cd1	2.431 (4)
O3—Cd1	2.476 (4)
O5—Cd1	2.305 (4)
N1—Cd1	2.407 (4)
N2—Cd1	2.359 (4)
N3—Cd1	2.393 (4)

**Table:** 

O5—Cd1—O1	150.47 (16)
O5—Cd1—N2	119.29 (16)
O1—Cd1—N2	88.94 (13)
O5—Cd1—N3	94.10 (16)
O1—Cd1—N3	87.79 (13)
N2—Cd1—N3	69.31 (13)
O5—Cd1—N1	106.73 (17)
O1—Cd1—N1	90.37 (14)
N2—Cd1—N1	69.73 (12)
N3—Cd1—N1	139.02 (13)
O5—Cd1—O2	77.34 (15)
O1—Cd1—O2	81.82 (14)
N2—Cd1—O2	149.55 (11)
N3—Cd1—O2	138.56 (14)
N1—Cd1—O2	81.32 (12)
O5—Cd1—O3	72.18 (16)
O1—Cd1—O3	78.57 (14)
N2—Cd1—O3	152.93 (12)
N3—Cd1—O3	86.13 (13)
N1—Cd1—O3	133.44 (13)
O2—Cd1—O3	52.52 (12)

**Table 2:** Hydrogen-bond geometry (Å, °)

*D*—H⋯*A*	*D*—H	H⋯*A*	*D*⋯*A*	*D*—H⋯*A*
O1—H1*B*⋯O3^i^	0.92 (5)	1.87 (5)	2.788 (5)	172

Symmetry code: (i) 
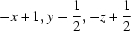
.

**Fig. 1.**
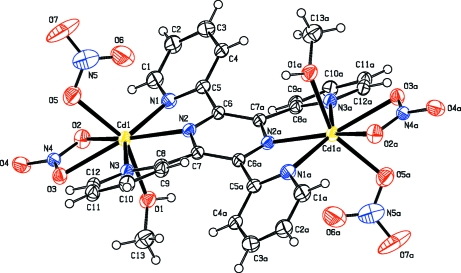
The molecular structure of the title molecule, with the atom-numbering scheme. Displacement ellipsoids are drawn at the 40% probability level [symmetry code: (a) 1 - x, -y, -z].

**Fig. 2.**
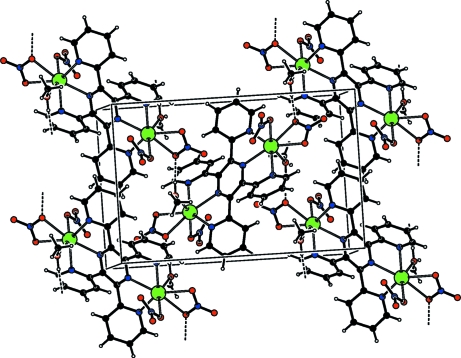
A packing diagram for the title molecule. Hydrogen bonds are shown as dashed lines.

## supplementary crystallographic information

### Comment

Goodwin & Lyons (1959) were reported the synthesis of 2,3,5,6-tetra- (2-pyridinyl)pyrazine (tppz). Bock *et al.* (1992) and Greaves & Stoeckli-Evans (1992) were determined the structure of tppz by single crystal X-ray analysis. Among many reported compounds containing tppz, most are complexes of transition metal ions, including ruthenium (Hadadzadeh *et al.*, 2006), platinum (Sakai & Kurashima, 2003), mercury (Zhang *et al.*, 2005), copper (Carranza *et al.*,  2004), iron (Laine *et al.*, 1995), nickel (Graf *et al.*,  1997), palladium (Yamada *et al.*,  2000) and zinc (Graf *et al.*,  1993). For further investigation of 2,3,5,6-tetra(2-pyridinyl)pyrazine, we synthesized the title complex, and report herein its crystal structure.

The title complex is a centrosymmetric dinuclear complex, in which the tppz ligand link two Cd ions separated by 7.323 (4) Å (Fig. 1). Each Cd^II^ center is seven-coordinated by three N donors of tppz in one plane, two O atoms (O1 and O5) nearly normal to this plane (Table 1) and two O atoms -0.393 (3) Å (for O2) and -0.488 (3) Å (for O3) away from that plane. The two Cd^II^ ions are above and below the plane of the pyrazine ring B (N2/C6/C7/N2a/C6a/C7a) [symmetry code: (a) 1 - x, -y, -z] of the tppz ligand. The dihedral angles between rings A (N1/C1-C5), B and C (N3/C8-C12) are A/B = 38.01 (3)°, A/C = 41.11 (3)° and B/C = 31.90 (3)°.

In the crystal structure, intermolecular O-H···O hydrogen bonds (Table 2) link the molecules (Fig. 2), in which they may be effective in the stabilization of the structure.

### Experimental

For the preparation of the title compound, a solution of 2,3,5,6-tetra- (2-pyridinyl)pyrazine (0.4 g, 1 mmol) in HCl_3_ (30 ml) was added to a solution of Cd(NO_3_)_2_.4H_2_O (0.62 g, 2 mmol) in methanol (200 ml) and the resulting colorless solution was stirred for 15 min at room temperature. Then, it was left to evaporate slowly at room temperature. After one week, colorless prismatic crystals of the title compound were isolated (yield; 0.71 g, 76.73%, m.p < 573 k).

### Refinement

H1B atom (for OH) was located in difference syntheses and refined isotropically [O-H = 0.92 (5) Å and U_iso_(H) = 0.020 (11) Å^2^]. The remaining H atoms were positioned geometrically, with C-H = 0.93 and 0.96 Å for aromatic and methyl H, respectively, and constrained to ride on their parent atoms with U_iso_(H) = 1.2U_eq_(C).

### Crystal data

**Table:**

[Cd_2_(NO_3_)_4_(C_24_H_16_N_6_)(CH_4_O)_2_]	*F*_000_ = 916
*M**_r_* = 925.37	*D*_x_ = 1.866 Mg m^−^^3^
Monoclinic, *P*2_1_/*c*	Mo *K*α radiation λ = 0.71073 Å
Hall symbol: -P 2ybc	Cell parameters from 1543 reflections
*a* = 9.0777 (12) Å	θ = 2.9–29.2º
*b* = 10.8949 (9) Å	µ = 1.38 mm^−^^1^
*c* = 16.690 (2) Å	*T* = 298 (2) K
β = 93.847 (10)º	Prism, colorless
*V* = 1646.9 (3) Å^3^	0.50 × 0.40 × 0.25 mm
*Z* = 2	

### Data collection

**Table:**

Stoe IPDSII diffractometer	4642 independent reflections
Radiation source: fine-focus sealed tube	4223 reflections with *I* > 2σ(*I*)
Monochromator: graphite	*R*_int_ = 0.075
Detector resolution: 0.15 mm pixels mm^-1^	θ_max_ = 29.8º
*T* = 298(2) K	θ_min_ = 2.9º
rotation method scans	*h* = −12→8
Absorption correction: numericalShape of crystal determined optically (X-SHAPE; Stoe & Cie, 2005)	*k* = −14→10
*T*_min_ = 0.510, *T*_max_ = 0.710	*l* = −22→19
4664 measured reflections	

### Refinement

**Table:**

Refinement on *F*^2^	Secondary atom site location: difference Fourier map
Least-squares matrix: full	Hydrogen site location: inferred from neighbouring sites
*R*[*F*^2^ > 2σ(*F*^2^)] = 0.062	H atoms treated by a mixture of independent and constrained refinement
*wR*(*F*^2^) = 0.171	*w* = 1/[σ^2^(*F*_o_^2^) + (0.1202*P*)^2^ + 2.8103*P*] where *P* = (*F*_o_^2^ + 2*F*_c_^2^)/3
*S* = 1.07	(Δ/σ)_max_ = 0.015
4642 reflections	Δρ_max_ = 1.00 e Å^−^^3^
239 parameters	Δρ_min_ = −0.95 e Å^−^^3^
Primary atom site location: structure-invariant direct methods	Extinction correction: none

### Special details

**Table:**

Geometry. All e.s.d.'s (except the e.s.d. in the dihedral angle between two l.s. planes) are estimated using the full covariance matrix. The cell e.s.d.'s are taken into account individually in the estimation of e.s.d.'s in distances, angles and torsion angles; correlations between e.s.d.'s in cell parameters are only used when they are defined by crystal symmetry. An approximate (isotropic) treatment of cell e.s.d.'s is used for estimating e.s.d.'s involving l.s. planes.
Refinement. Refinement of F^2^ against ALL reflections. The weighted R-factor wR and goodness of fit S are based on F^2^, conventional R-factors R are based on F, with F set to zero for negative F^2^. The threshold expression of F^2^ > 2sigma(F^2^) is used only for calculating R-factors(gt) etc. and is not relevant to the choice of reflections for refinement. R-factors based on F^2^ are statistically about twice as large as those based on F, and R- factors based on ALL data will be even larger.

### Fractional atomic coordinates and isotropic or equivalent isotropic displacement parameters (Å^2^)

**Table:**

	*x*	*y*	*z*	*U*_iso_*/*U*_eq_	
Cd1	0.38914 (3)	0.18792 (3)	0.167582 (18)	0.02737 (16)	
O1	0.5852 (4)	0.0852 (3)	0.2353 (3)	0.0397 (8)	
H1B	0.580 (6)	0.001 (5)	0.233 (3)	0.020 (11)*	
O2	0.2968 (5)	0.1892 (3)	0.3010 (3)	0.0391 (9)	
O3	0.4556 (4)	0.3323 (3)	0.2788 (2)	0.0359 (8)	
O4	0.3358 (5)	0.3363 (5)	0.3875 (3)	0.0502 (10)	
O5	0.2209 (6)	0.3467 (5)	0.1596 (3)	0.0541 (10)	
O6	0.1241 (9)	0.2195 (8)	0.0768 (8)	0.118 (4)	
O7	−0.0039 (8)	0.3728 (10)	0.1114 (5)	0.115 (3)	
N1	0.2489 (4)	0.0003 (4)	0.1585 (3)	0.0343 (8)	
N2	0.4388 (4)	0.0798 (3)	0.0499 (2)	0.0270 (7)	
N3	0.5671 (4)	0.2952 (3)	0.0946 (3)	0.0295 (7)	
N4	0.3612 (4)	0.2867 (4)	0.3234 (2)	0.0288 (7)	
N5	0.1090 (6)	0.3123 (4)	0.1154 (4)	0.0449 (12)	
C1	0.1339 (6)	−0.0214 (5)	0.2045 (4)	0.0440 (12)	
H1	0.1363	0.0123	0.2558	0.053*	
C2	0.0136 (6)	−0.0917 (6)	0.1781 (4)	0.0482 (14)	
H2	−0.0620	−0.1068	0.2117	0.058*	
C3	0.0071 (5)	−0.1392 (6)	0.1013 (4)	0.0465 (13)	
H3	−0.0750	−0.1838	0.0816	0.056*	
C4	0.1251 (5)	−0.1196 (5)	0.0536 (3)	0.0365 (10)	
H4	0.1232	−0.1502	0.0016	0.044*	
C5	0.2455 (4)	−0.0533 (4)	0.0855 (3)	0.0314 (8)	
C6	0.3792 (4)	−0.0321 (4)	0.0401 (3)	0.0264 (8)	
C7	0.5546 (4)	0.1162 (4)	0.0106 (3)	0.0258 (7)	
C8	0.5956 (4)	0.2482 (4)	0.0224 (3)	0.0279 (8)	
C9	0.6505 (5)	0.3192 (4)	−0.0380 (3)	0.0319 (10)	
H9	0.6588	0.2868	−0.0890	0.038*	
C10	0.6926 (5)	0.4390 (4)	−0.0209 (3)	0.0361 (10)	
H10	0.7325	0.4875	−0.0599	0.043*	
C11	0.6746 (5)	0.4859 (4)	0.0553 (3)	0.0358 (10)	
H11	0.7062	0.5648	0.0689	0.043*	
C12	0.6086 (5)	0.4124 (4)	0.1104 (3)	0.0324 (9)	
H12	0.5920	0.4451	0.1605	0.039*	
C13	0.7367 (7)	0.1300 (6)	0.2397 (5)	0.0525 (14)	
H13A	0.7409	0.2093	0.2650	0.063*	
H13B	0.7702	0.1366	0.1865	0.063*	
H13C	0.7990	0.0738	0.2706	0.063*	

### Atomic displacement parameters (Å^2^)

**Table:**

	*U*^11^	*U*^22^	*U*^33^	*U*^12^	*U*^13^	*U*^23^
Cd1	0.0283 (2)	0.0260 (2)	0.0286 (2)	0.00137 (9)	0.00796 (15)	−0.00333 (9)
O1	0.0364 (16)	0.0306 (16)	0.052 (2)	0.0038 (13)	0.0046 (16)	0.0021 (15)
O2	0.045 (2)	0.0310 (17)	0.043 (2)	−0.0054 (12)	0.0127 (18)	0.0033 (13)
O3	0.0322 (15)	0.0335 (14)	0.043 (2)	−0.0044 (12)	0.0110 (16)	−0.0079 (14)
O4	0.0327 (17)	0.082 (3)	0.037 (2)	−0.0043 (18)	0.0072 (16)	−0.023 (2)
O5	0.056 (2)	0.056 (2)	0.050 (2)	0.018 (2)	−0.002 (2)	−0.004 (2)
O6	0.090 (5)	0.076 (4)	0.179 (11)	0.014 (4)	−0.058 (6)	−0.043 (6)
O7	0.063 (4)	0.176 (8)	0.107 (6)	0.073 (5)	0.014 (4)	0.038 (6)
N1	0.0264 (16)	0.042 (2)	0.0352 (19)	−0.0026 (14)	0.0116 (16)	−0.0054 (16)
N2	0.0235 (14)	0.0275 (15)	0.0303 (17)	0.0017 (12)	0.0046 (14)	−0.0009 (13)
N3	0.0296 (17)	0.0284 (15)	0.0311 (18)	0.0012 (13)	0.0064 (15)	−0.0029 (14)
N4	0.0234 (15)	0.0354 (16)	0.0279 (17)	−0.0004 (13)	0.0047 (14)	−0.0077 (15)
N5	0.051 (2)	0.091 (3)	0.082 (3)	0.0054 (16)	0.002 (2)	0.015 (2)
C1	0.032 (2)	0.053 (3)	0.050 (3)	−0.005 (2)	0.018 (2)	−0.011 (2)
C2	0.031 (2)	0.055 (3)	0.060 (3)	−0.009 (2)	0.021 (2)	−0.015 (3)
C3	0.028 (2)	0.052 (3)	0.061 (3)	−0.009 (2)	0.012 (2)	−0.013 (3)
C4	0.0243 (17)	0.039 (2)	0.047 (3)	0.0025 (16)	0.0053 (18)	−0.012 (2)
C5	0.0228 (16)	0.036 (2)	0.036 (2)	0.0003 (15)	0.0055 (16)	−0.0057 (18)
C6	0.0229 (16)	0.0293 (18)	0.0275 (18)	0.0006 (13)	0.0050 (15)	−0.0047 (15)
C7	0.0219 (15)	0.0279 (17)	0.0282 (18)	0.0002 (14)	0.0050 (15)	−0.0029 (15)
C8	0.0230 (16)	0.0288 (18)	0.033 (2)	0.0007 (14)	0.0063 (15)	−0.0021 (16)
C9	0.0276 (19)	0.032 (2)	0.037 (2)	−0.0010 (14)	0.0117 (19)	−0.0024 (16)
C10	0.034 (2)	0.032 (2)	0.044 (2)	−0.0045 (17)	0.012 (2)	−0.0003 (19)
C11	0.033 (2)	0.0295 (19)	0.046 (3)	−0.0027 (16)	0.009 (2)	−0.0044 (19)
C12	0.0320 (18)	0.0294 (19)	0.036 (2)	0.0036 (15)	0.0055 (18)	−0.0053 (17)
C13	0.044 (3)	0.051 (3)	0.063 (4)	0.003 (2)	0.008 (3)	0.005 (3)

### Geometric parameters (Å, °)

**Table:**

O1—Cd1	2.330 (4)	C4—H4	0.9300
O1—H1B	0.92 (5)	C5—N1	1.349 (6)
O2—Cd1	2.431 (4)	C5—C6	1.492 (5)
O3—Cd1	2.476 (4)	C6—N2	1.340 (5)
O5—Cd1	2.305 (4)	C6—C7^i^	1.409 (5)
N1—Cd1	2.407 (4)	C7—N2	1.336 (5)
N2—Cd1	2.359 (4)	C7—C6^i^	1.409 (5)
N3—Cd1	2.393 (4)	C7—C8	1.495 (6)
N4—O4	1.234 (5)	C8—N3	1.350 (6)
N4—O2	1.257 (5)	C8—C9	1.389 (6)
N4—O3	1.273 (5)	C9—C10	1.385 (6)
N5—O6	1.211 (10)	C9—H9	0.9300
N5—O7	1.217 (7)	C10—C11	1.390 (7)
N5—O5	1.271 (8)	C10—H10	0.9300
C1—N1	1.357 (6)	C11—C12	1.387 (6)
C1—C2	1.382 (8)	C11—H11	0.9300
C1—H1	0.9300	C12—N3	1.352 (6)
C2—C3	1.380 (9)	C12—H12	0.9300
C2—H2	0.9300	C13—O1	1.457 (7)
C3—C4	1.393 (6)	C13—H13A	0.9600
C3—H3	0.9300	C13—H13B	0.9600
C4—C5	1.385 (6)	C13—H13C	0.9600
			
O5—Cd1—O1	150.47 (16)	N1—C1—C2	122.5 (5)
O5—Cd1—N2	119.29 (16)	N1—C1—H1	118.8
O1—Cd1—N2	88.94 (13)	C2—C1—H1	118.8
O5—Cd1—N3	94.10 (16)	C3—C2—C1	119.2 (5)
O1—Cd1—N3	87.79 (13)	C3—C2—H2	120.4
N2—Cd1—N3	69.31 (13)	C1—C2—H2	120.4
O5—Cd1—N1	106.73 (17)	C2—C3—C4	119.0 (5)
O1—Cd1—N1	90.37 (14)	C2—C3—H3	120.5
N2—Cd1—N1	69.73 (12)	C4—C3—H3	120.5
N3—Cd1—N1	139.02 (13)	C5—C4—C3	118.5 (5)
O5—Cd1—O2	77.34 (15)	C5—C4—H4	120.7
O1—Cd1—O2	81.82 (14)	C3—C4—H4	120.7
N2—Cd1—O2	149.55 (11)	N1—C5—C4	122.8 (4)
N3—Cd1—O2	138.56 (14)	N1—C5—C6	114.9 (4)
N1—Cd1—O2	81.32 (12)	C4—C5—C6	122.2 (4)
O5—Cd1—O3	72.18 (16)	N2—C6—C7^i^	118.7 (3)
O1—Cd1—O3	78.57 (14)	N2—C6—C5	114.5 (3)
N2—Cd1—O3	152.93 (12)	C7^i^—C6—C5	126.8 (4)
N3—Cd1—O3	86.13 (13)	N2—C7—C6^i^	118.9 (4)
N1—Cd1—O3	133.44 (13)	N2—C7—C8	114.7 (3)
O2—Cd1—O3	52.52 (12)	C6^i^—C7—C8	126.3 (3)
C13—O1—Cd1	123.5 (3)	N3—C8—C9	122.4 (4)
C13—O1—H1B	112 (3)	N3—C8—C7	114.9 (3)
Cd1—O1—H1B	115 (4)	C9—C8—C7	122.6 (4)
N4—O2—Cd1	95.5 (3)	C10—C9—C8	118.9 (5)
N4—O3—Cd1	92.9 (2)	C10—C9—H9	120.6
N5—O5—Cd1	108.2 (4)	C8—C9—H9	120.6
C5—N1—C1	117.6 (4)	C9—C10—C11	119.1 (4)
C5—N1—Cd1	113.9 (3)	C9—C10—H10	120.4
C1—N1—Cd1	122.4 (3)	C11—C10—H10	120.4
C7—N2—C6	122.3 (3)	C12—C11—C10	118.5 (4)
C7—N2—Cd1	117.5 (3)	C12—C11—H11	120.7
C6—N2—Cd1	117.3 (3)	C10—C11—H11	120.7
C8—N3—C12	117.7 (4)	N3—C12—C11	122.8 (4)
C8—N3—Cd1	116.4 (3)	N3—C12—H12	118.6
C12—N3—Cd1	123.4 (3)	C11—C12—H12	118.6
O4—N4—O2	121.2 (4)	O1—C13—H13A	109.5
O4—N4—O3	120.5 (4)	O1—C13—H13B	109.5
O2—N4—O3	118.2 (4)	H13A—C13—H13B	109.5
O6—N5—O7	123.2 (9)	O1—C13—H13C	109.5
O6—N5—O5	116.1 (6)	H13A—C13—H13C	109.5
O7—N5—O5	120.6 (8)	H13B—C13—H13C	109.5
			
N1—C1—C2—C3	−1.9 (10)	C13—O1—Cd1—N3	19.8 (4)
C1—C2—C3—C4	2.8 (10)	C13—O1—Cd1—N1	158.9 (4)
C2—C3—C4—C5	0.4 (9)	C13—O1—Cd1—O2	−119.9 (4)
C3—C4—C5—N1	−4.9 (8)	C13—O1—Cd1—O3	−66.7 (4)
C3—C4—C5—C6	177.5 (5)	C7—N2—Cd1—O5	94.7 (4)
N1—C5—C6—N2	−36.5 (6)	C6—N2—Cd1—O5	−104.3 (4)
C4—C5—C6—N2	141.4 (5)	C7—N2—Cd1—O1	−76.2 (3)
N1—C5—C6—C7^i^	143.9 (5)	C6—N2—Cd1—O1	84.8 (3)
C4—C5—C6—C7^i^	−38.2 (7)	C7—N2—Cd1—N3	11.8 (3)
N2—C7—C8—N3	31.1 (6)	C6—N2—Cd1—N3	172.8 (4)
C6^i^—C7—C8—N3	−152.5 (4)	C7—N2—Cd1—N1	−167.0 (4)
N2—C7—C8—C9	−145.2 (4)	C6—N2—Cd1—N1	−6.0 (3)
C6^i^—C7—C8—C9	31.2 (7)	C7—N2—Cd1—O2	−148.0 (3)
N3—C8—C9—C10	7.2 (7)	C6—N2—Cd1—O2	13.0 (5)
C7—C8—C9—C10	−176.8 (5)	C7—N2—Cd1—O3	−14.4 (5)
C8—C9—C10—C11	−2.0 (8)	C6—N2—Cd1—O3	146.6 (3)
C9—C10—C11—C12	−2.9 (8)	C8—N3—Cd1—O5	−114.3 (3)
C10—C11—C12—N3	3.1 (8)	C12—N3—Cd1—O5	47.6 (4)
C4—C5—N1—C1	5.8 (8)	C8—N3—Cd1—O1	95.3 (3)
C6—C5—N1—C1	−176.4 (5)	C12—N3—Cd1—O1	−102.9 (4)
C4—C5—N1—Cd1	−147.4 (4)	C8—N3—Cd1—N2	5.6 (3)
C6—C5—N1—Cd1	30.5 (5)	C12—N3—Cd1—N2	167.4 (4)
C2—C1—N1—C5	−2.4 (9)	C8—N3—Cd1—N1	7.2 (5)
C2—C1—N1—Cd1	148.4 (5)	C12—N3—Cd1—N1	169.1 (4)
C6^i^—C7—N2—C6	−3.0 (7)	C8—N3—Cd1—O2	170.3 (3)
C8—C7—N2—C6	173.7 (4)	C12—N3—Cd1—O2	−27.9 (5)
C6^i^—C7—N2—Cd1	157.0 (3)	C8—N3—Cd1—O3	173.9 (3)
C8—C7—N2—Cd1	−26.3 (5)	C12—N3—Cd1—O3	−24.2 (4)
C7^i^—C6—N2—C7	3.0 (7)	C5—N1—Cd1—O5	102.0 (3)
C5—C6—N2—C7	−176.7 (4)	C1—N1—Cd1—O5	−49.8 (5)
C7^i^—C6—N2—Cd1	−157.0 (3)	C5—N1—Cd1—O1	−102.5 (3)
C5—C6—N2—Cd1	23.3 (5)	C1—N1—Cd1—O1	105.8 (5)
C9—C8—N3—C12	−7.0 (7)	C5—N1—Cd1—N2	−13.7 (3)
C7—C8—N3—C12	176.7 (4)	C1—N1—Cd1—N2	−165.5 (5)
C9—C8—N3—Cd1	155.9 (4)	C5—N1—Cd1—N3	−15.4 (5)
C7—C8—N3—Cd1	−20.4 (5)	C1—N1—Cd1—N3	−167.2 (4)
C11—C12—N3—C8	1.7 (7)	C5—N1—Cd1—O2	175.9 (4)
C11—C12—N3—Cd1	−159.9 (4)	C1—N1—Cd1—O2	24.1 (5)
O4—N4—O2—Cd1	171.9 (4)	C5—N1—Cd1—O3	−177.0 (3)
O3—N4—O2—Cd1	−9.3 (5)	C1—N1—Cd1—O3	31.3 (5)
O4—N4—O3—Cd1	−172.1 (4)	N4—O2—Cd1—O5	−71.8 (3)
O2—N4—O3—Cd1	9.1 (5)	N4—O2—Cd1—O1	87.1 (3)
O6—N5—O5—Cd1	−12.9 (9)	N4—O2—Cd1—N2	160.8 (3)
O7—N5—O5—Cd1	169.2 (6)	N4—O2—Cd1—N3	9.9 (4)
N5—O5—Cd1—O1	−154.5 (3)	N4—O2—Cd1—N1	178.8 (3)
N5—O5—Cd1—N2	44.2 (4)	N4—O2—Cd1—O3	5.3 (3)
N5—O5—Cd1—N3	112.8 (4)	N4—O3—Cd1—O5	82.3 (3)
N5—O5—Cd1—N1	−31.5 (4)	N4—O3—Cd1—O1	−93.6 (3)
N5—O5—Cd1—O2	−108.3 (4)	N4—O3—Cd1—N2	−157.7 (3)
N5—O5—Cd1—O3	−162.6 (4)	N4—O3—Cd1—N3	177.9 (3)
C13—O1—Cd1—O5	−74.6 (5)	N4—O3—Cd1—N1	−14.1 (4)
C13—O1—Cd1—N2	89.2 (4)	N4—O3—Cd1—O2	−5.2 (3)

Symmetry codes: (i) −*x*+1, −*y*, −*z*.

### Hydrogen-bond geometry (Å, °)

**Table:**

*D*—H···*A*	*D*—H	H···*A*	*D*···*A*	*D*—H···*A*
O1—H1B···O3^ii^	0.92 (5)	1.87 (5)	2.788 (5)	172

Symmetry codes: (ii) −*x*+1, *y*−1/2, −*z*+1/2.
